# Effects of neural progenitor cells on post-stroke neurological impairment—a detailed and comprehensive analysis of behavioral tests

**DOI:** 10.3389/fncel.2014.00338

**Published:** 2014-10-22

**Authors:** Thorsten R. Doeppner, Britta Kaltwasser, Mathias Bähr, Dirk M. Hermann

**Affiliations:** ^1^Department of Neurology, University of Duisburg-Essen Medical SchoolEssen, Germany; ^2^Department of Neurology, University of Goettingen Medical SchoolGoettingen, Germany

**Keywords:** cerebral ischemia, behavioral tests, neural progenitor cells, stroke, functional outcome, stem cells, transplantation

## Abstract

Systemic transplantation of neural progenitor cells (NPCs) in rodents reduces functional impairment after cerebral ischemia. In light of upcoming stroke trials regarding safety and feasibility of NPC transplantation, experimental studies have to successfully analyze the extent of NPC-induced neurorestoration on the functional level. However, appropriate behavioral tests for analysis of post-stroke motor coordination deficits and cognitive impairment after NPC grafting are not fully established. We therefore exposed male C57BL6 mice to either 45 min (mild) or 90 min (severe) of cerebral ischemia, using the thread occlusion model followed by intravenous injection of PBS or NPCs 6 h post-stroke with an observation period of three months. Post-stroke motor coordination was assessed by means of the rota rod, tight rope, corner turn, inclined plane, grip strength, foot fault, adhesive removal, pole test and balance beam test, whereas cognitive impairment was analyzed using the water maze, the open field and the passive avoidance test. Significant motor coordination differences after both mild and severe cerebral ischemia in favor of NPC-treated mice were observed for each motor coordination test except for the inclined plane and the grip strength test, which only showed significant differences after severe cerebral ischemia. Cognitive impairment after mild cerebral ischemia was successfully assessed using the water maze test, the open field and the passive avoidance test. On the contrary, the water maze test was not suitable in the severe cerebral ischemia paradigm, as it too much depends on motor coordination capabilities of test mice. In terms of both reliability and cost-effectiveness considerations, we thus recommend the corner turn, foot fault, balance beam, and open field test, which do not depend on durations of cerebral ischemia.

## Introduction

Stem cells or progenitor cells from various tissue sources reduce both brain injury and functional impairment after cerebral ischemia (Bliss et al., 2007, 2010; Bacigaluppi et al., 2008, 2009; Schwarting et al., 2008; Doeppner et al., 2010; Zheng et al., 2010; Leong et al., 2012). Despite recent progress using induced neural stem cells in a non-ischemic mouse model (Hemmer et al., 2014), grafted cells are generally not thought to be integrated into the residing post-ischemic neural network. Rather, the aforementioned beneficial effects due to cell transplantation are attributed to indirect effects independent of genuine cell replacement. With the therapeutic use of both embryonic stem cells and multipotent stem cells restricted due to ethical concerns and tumor formation (Blum and Benvenisty, 2008, 2009), application of adult stem/progenitor cells like neural progenitor cells (NPCs) has become a feasible therapeutic approach in experimental stroke research (Bonnamain et al., 2012).

Since endogenous neurogenesis persists within distinct regions of the adult rodent brain such as the subventricular zone of the lateral ventricles (Alvarez-Buylla and Garcia-Verdugo, 2002), NPCs can be harvested from these areas and transferred to cell culture (Reynolds and Weiss, 1992). Upon *in vitro* expansion, cultivated NPCs are used for both systemic and local cell transplantation studies in various stroke models. Transplantation of NPCs using either systemic or local cell delivery routes reduces post-ischemic injury (Mochizuki et al., 2008; Bacigaluppi et al., 2009; Hicks et al., 2009; Doeppner et al., 2010, 2012a). With regard to clinical relevance, however, intravenous transplantation of NPCs is more feasible than stereotactic intracranial cell delivery.

Although original work as well as meta-analyses regarding assessment of functional outcome in both ischemic and non-ischemic rodents do exist (Gerlai et al., 2000; Li et al., 2004; Bouët et al., 2007; Brooks and Dunnett, 2009; Schaar et al., 2010; Balkaya et al., 2012), original work that systematically studies post-stroke functional outcome after transplantation of NPCs or other stem cells on a wide variety of behavioral tests does not exist. As a matter of fact, the majority of the aforementioned experimental studies evaluating post-stroke functional outcome after cell transplantation are limited to the application of only a few behavioral tests. However, in view of clinical stroke trials applying NPC transplantation already on the way (NCT02117635 and NCT01151124) and in respect of further trials in the future, reliable and valid experimental data regarding the feasibility of NPCs in stroke treatment is vital in order to avoid negative clinical trials. Therefore, we systematically analyzed both motor coordination deficits and cognitive impairment after intravenous transplantation of NPCs for as long as three months post-stroke using a total of twelve behavioral tests. Since correlation between infarct volumes and functional outcome is certain for at least some behavioral tests such as the cylinder test and the grid walk test (Rogers et al., 1997), we used two experimental paradigms by exposing animals to either mild (45 min) or severe (90 min) cerebral ischemia followed by intravenous transplantation of NPCs 6 h post-stroke. By excluding impacts due to different laboratories and experimental modalities, our study might give some advice for studying post-stroke functional outcome after cell transplantation in rodents and thus set the path for future clinical trials.

## Materials and methods

### Experimental paradigm and animals used

**Figure 1:**
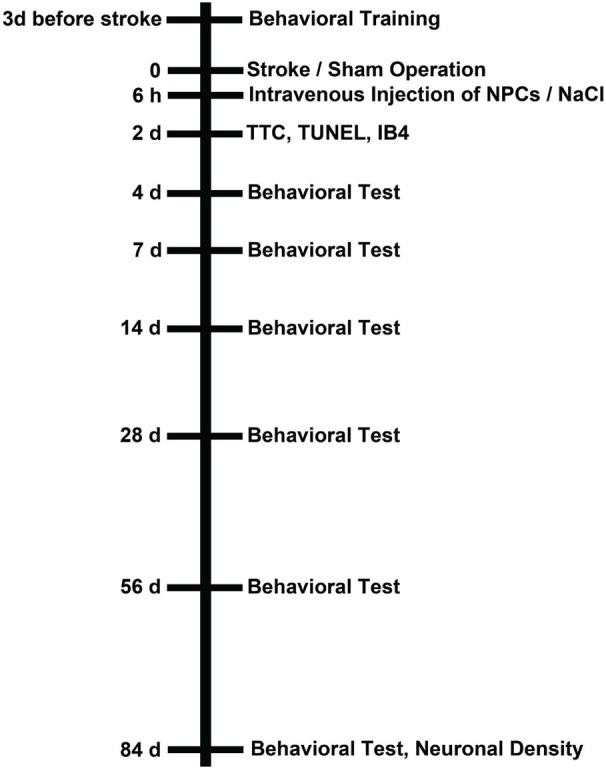
**Experimental paradigm**. Male C57BL6 mice were exposed to either 45 min or 90 min of cerebral ischemia followed by survival periods of either 2 days or 84 days. Sham animals underwent the same operational technique, but without actually inserting a filament for occlusion of the left middle cerebral artery. All mice received an intravenous injection of PBS or (NPCs; 10^6^ cells in 100 µl of PBS) via cannulation of the femoral vein 6 h after stroke (or sham) operation. Those animals that were sacrificed on day 2 after stroke/sham operation were used for assessment of infarct volumes and determination of TUNEL^+^ cells as well as microglial activity using IB4 staining. Mice that were allowed to survive for up to 84 days were used for behavioral tests at the time points given as well as for analysis of neuronal density on day 84. All mice (including sham animals) were trained for behavioral tests on days 1–3 before induction of stroke or sham operation.

All experimental procedures were performed according to the guidelines for the care and use of laboratory animals and were approved by local authorities, i.e., LANUV North-Rhine-Westphalia and Government of Lower Saxony. Both experiments and analyses of data were blinded to experimenters. For all experiments male C57BL6 (10–12 weeks old, 23–25 g; Charles River, Germany) mice were used that had free access to food and water. Mice were exposed to either 45 min or 90 min of cerebral ischemia as mentioned below. The animals were allowed to survive for 2 days (analysis of brain injury) or for 84 days (behavioral days and assessment of brain injury) post-stroke. Three experimental groups were defined for each condition, i.e., time point (2 days vs. 84 days) and duration of cerebral ischemia (45 min vs. 90 min). These groups consisted of mice treated with intravenous injections of NPCs or PBS at 6 h post-stroke and of sham mice. The latter underwent middle cerebral artery “occlusion” (MCAO) without actually inserting a filament, but were intravenously injected with PBS at 6 h post-stroke. The number of animals used for the 2-day survival was six for each condition with a survival rate of 100%. Since mice used for infarct volume analysis could not be used for immunohistochemistry at the same time, the total number of animals used for this time point was 72. The number of mice used for statistical analysis for the 84-day time point was as follows. For the mild cerebral ischemia paradigm, 15 sham animals (survival rate 100%), 14 NPC-treated mice (survival rate 93.3%) and 16 controls (survival rate 88.8%) were used for statistical analysis. For the severe cerebral ischemia paradigm, 15 sham animals (survival rate 100%), 16 NPC-treated mice (survival rate 84.2%), and 17 controls (survival rate 80.9%) were used. Noteworthy, some mice suffering from severe cerebral ischemia could not perform all behavioral tests due to the severity of the disease. When applicable, this is explicitly mentioned in the results part and the corresponding figure legend as well. All mice that were allowed to survive for 84 days post-stroke were used for immunohistochemistry (neuronal density) and behavioral analysis. For the latter purpose, mice were trained for each behavioral test on days 3–1 before induction of cerebral ischemia. An overview of the experimental paradigm is given in Figure 1.

### Induction of cerebral ischemia

Transient focal cerebral ischemia was performed as described before (Doeppner et al., 2012b). Stroke was induced using the MCAO model. Briefly, mice underwent anesthesia by means of isoflurane (1–1.5%, 30% O_2_, remainder N_2_) under constant control of body temperature using a rectal thermometer with a feedback control system. Moreover, cerebral blood flow was constantly controlled using a Laser Doppler probe (Perimed, Sweden) that was attached to the skull. MCAO itself was achieved using a 7–0 silicon rubber coated monofilament (coating length 4–5 mm, tip diameter 180 mm; Doccol, USA) that was inserted for either 45 min or 90 min in order to induce stroke. Sham operated mice received the same operation except for inserting a filament, but were kept under anesthesia for 45 min or 90 min to match corresponding periods of cerebral ischemia.

### Cultivation and transplantation of neural progenitor cells (NPCs)

Preparation of SVZ-derived NPCs was performed as previously described (Doeppner et al., 2012a). Briefly, NPCs were isolated from the SVZ of 6–8 week old male transgenic green fluorescence protein positive animals (C57BL/6-Tg ACTB-enhanced green fluorescence protein (EGFP), 1Osb/J; JAX Laboratory, Bar Harbor, USA). EGFP expression was under control of the actin promoter, thus providing reliable and stable tracking of grafted NPCs. The prepared EGFP^+^ NPCs were cultured in serum-free basic Dulbecco’s modified Eagle’s medium (DMEM)/F12 (PAA, Linz, Austria) supplemented with epidermal growth factor (EGF, 2 µg/ml), basic fibroblast growth factor (bFGF, 2 µg/ml), and penicillin-streptomycin (Invitrogen, Frankfurt, Germany). Cells were incubated with 5% CO_2_ at 37°C, and growth factors were added every 2–3 days. Passaging of cells was done every 7–10 days and NPCs used for transplantation were from cell passages 3–6.

The NPCs were intravenously transplanted 6 h after stroke, a time point that has previously been shown by us to be associated with post-ischemic neuroprotection after mild cerebral ischemia (Doeppner et al., 2012a). The right femoral vein was cannulated under deep anesthesia, and 10^6^ NPCs (solved in 100 µl PBS) were injected over 10 min. Controls and sham operated mice received 100 µl of PBS over 10 min.

### Assessment of brain injury

Brain injury on day 2 post-stroke was assessed via determination of infarct volumes as well as via analysis of terminal deoxynucleotidyl transferase (TdT)-mediated dUTP nick end labeling (TUNEL) and microglial activity. Infarct volume analysis was done using a staining with 2,3,5-triphenyltetrazolium chloride (TTC; 2%). Brains were removed and cut into slices of 2 mm each followed by TTC staining and a computer-based analysis of infarct volumes using the freely available software ImageJ.

Immunohistochemical analyses were performed on days 2 and 84. Mice received i.p. injections with chloralhydrate (420 mg/kg body weight) at the time points given and were transcardially perfused with 4% paraformaldehyde. Thereafter, the brains were removed, shock-frozen in liquid nitrogen, and 16-µm thick coronal cryostat sections were prepared. For quantitative analyses, a region of interest (ROIs) within the ischemic basal ganglia was defined in which three microscopic fields were set. The size of each microscopic field was 0.25 mm^2^. Quantitative analysis was performed using a 20 × magnification. The stereotactic coordinates for the definition of the ROI was 0.14 mm anterior, 2.5–3.25 mm ventral and 1.5–2.25 mm lateral from bregma. A total of three sections per condition were analyzed in the aforementioned manner.

For TUNEL staining on day 2, sections were incubated with proteinase K (7 min at 37°C) followed by exposure to the TdT enzyme reaction according to the manufacturer’s manual (Roche, Switzerland). Thereafter, sections were stained with a streptavidin-Alexa-594-conjugated secondary antibody (2 h at room temperature; Invitrogen, Germany) and analyzed. Microglial activity within the aforementioned ROIs was done on day 2 using a rat biotin-conjugated anti-IB4 antibody (1:100; Vector, UK). After 18 h of incubation at 4°C and several washing steps, a Streptavidin-Alexa 488 antibody (1:500; Molecular Probes, Germany) was used as secondary antibody. Long-term post-ischemic brain injury was performed by means of analysis of neuronal density. Therefore, NeuN staining was done on day 84 post-stroke. NeuN staining was performed using a mouse monoclonal anti-NeuN antibody (1:200; Chemicon, UK; 18 h, 4°C) and a mouse anti-mouse Alexa 488 (1:400; Molecular Probes) as secondary antibody. Quantitative analysis was achieved using the above mentioned ROI.

### Adhesive removal test

The test analyzes both forepaw sensitivity including presence of neglect as well as motor impairments of the forepaw. This test was performed as previously described (Bouët et al., 2007). Briefly, mice were placed in a transparent box (150 cm^2^) for 1 min in order to allow habituation. Thereafter, the mouse was taken out of the box and one adhesive tape (0.3 × 0.4 cm) was attached on each mouse paw. The order of attaching the tape (right or left) was changed between different trials and different animals alike. Thereafter, the mouse was put into the box and the time to either contact and to remove the tape was analyzed for a total of 120 s. Data for analysis of the contralateral (i.e., impaired) forepaw are shown in Figures 3, 5, whereas data for the ipsilateral (i.e., non-impaired) forepaw are shown in supplementary Figure S1.

**Figure 2:**
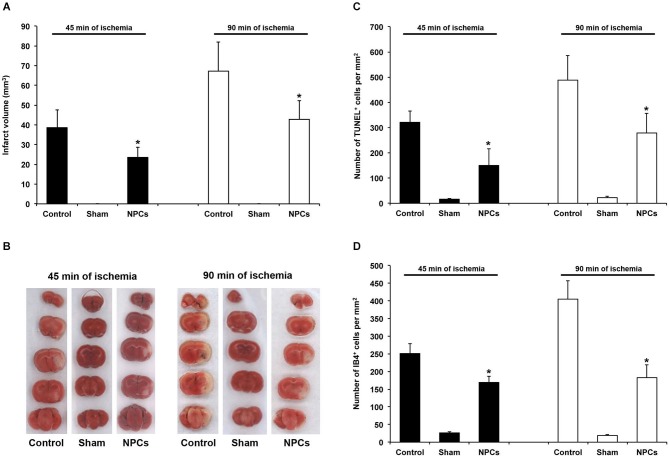
**NPCs induce acute neuroprotection after both mild and severe transient focal cerebral ischemia**. PBS or neural progenitor cells (NPCs) were given 6 h after stroke induction. Sham mice underwent stroke operation without inserting the filament into the left middle cerebral artery. In order to avoid effects of anesthesia and allow proper comparison to NPC-treated animals, sham mice were kept for either 45 min or 90 min under anesthesia corresponding to stroke periods of NPC-treated mice or controls. All sham animals received additional anesthesia 6 h after “stroke” operation with cannulation of the right femoral vein and injection of PBS. **(A)** Infarct analysis on day 2 after induction of cerebral ischemia for either 45 min or 90 min using TTC staining. **(B)** Representative TTC stainings for each experimental group. **(C)** Analysis of TUNEL^+^ cells on day 2 for each experimental group. **(D)** Quantitative analysis of IB4^+^ cells within the ischemic striatum on day 2 post-stroke, using the same experimental paradigm as mentioned above. Number of animals used was *n* = 6 for each experimental paradigm. * Significantly different from control, *p* < 0.05.

**Figure 3:**
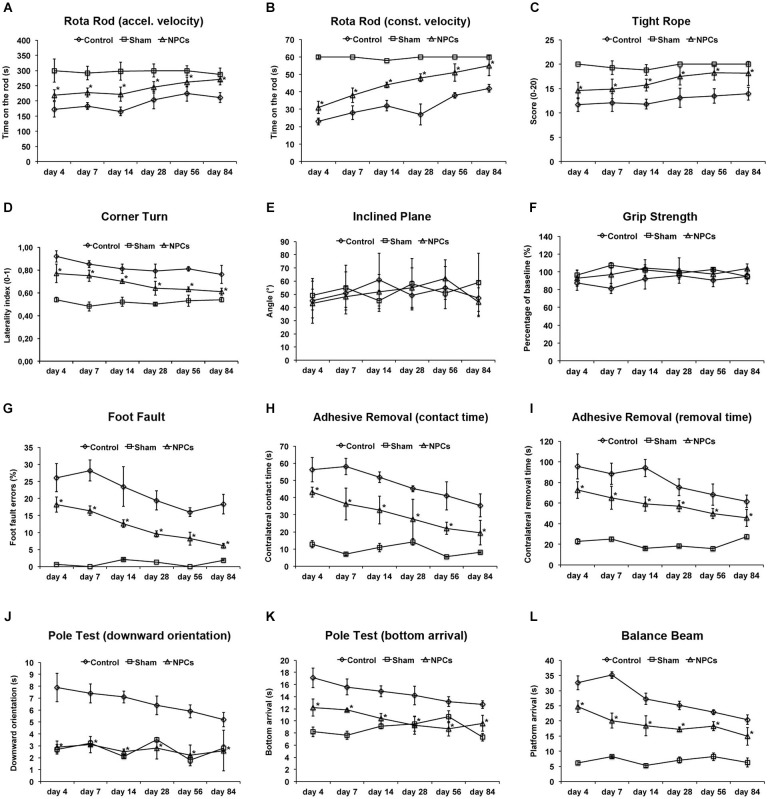
**Neural progenitor cells reduce motor coordination impairment after mild cerebral ischemia**. Mice were exposed to mild cerebral ischemia (45 min) and allowed to survive for 84 days. Animals received intravenous injection of either neural progenitor cells (“NPCs”) or PBS (“Control”) 6 h after stroke. Sham animals (“Sham”) underwent the same operational technique, but without inserting a filament followed by intravenous injection of 100 µl PBS at 6 h. Behavioral tests included the rota rod test with accelerating **(A)** and constant **(B)** velocity, for which maximal testing time was 300 s **(A)** and 60 s **(B)**. For the tight rope test **(C)**, a validated score was used from 0 (min) to 20 (max). The corner turn test **(D)** was analyzed using the laterality index (number of left turns – number of right turns)/10. For the inclined plain test **(E)**, the angle until the mice was still able to hold its ground was measured and used for statistical analysis, whereas the grip strength test **(F)** analyzed the percentage of force an animal was able to exert on a steel wire. For the foot fault test **(G)**, mice were placed on an elevated steel grid, and the relative number of foot faults of the right (impaired) forelimb was counted. The adhesive removal test included attachment of a defined tape at the paws with subsequent determination of both contact time **(H)** and removal time **(I)**. Maximal testing time of the test was 120 s. The pole test analyzed both downward orientation **(J)** and bottom arrival **(K)** when the mouse was placed head upward on the top of a vertical pole, thus predominantly testing extrapyramidal motor locomotion. Maximal testing time was 60 s for these tests. If the animal was unsuccessful for either task, it was scored 60 s. The balance beam test **(L)** consisted of an apparatus with a 110 cm long beam with constantly reduced width, starting with 12 mm width at the beginning and 5 mm width at the end of the beam. The time (maximal time was 60 s) until the mouse reached the platform at the end of the beam was measured. Maximal testing time was 60 s. The tests were performed at the time points given for as long as 84 days post-stroke as described in the materials and methods section of the manuscript. Number of animals used for statistical analysis was *n* = 15 for sham animals, *n* = 14 for NPC-treated mice and *n* = 16 for controls. * Significantly different from control, *p* < 0.05.

**Figure 4:**
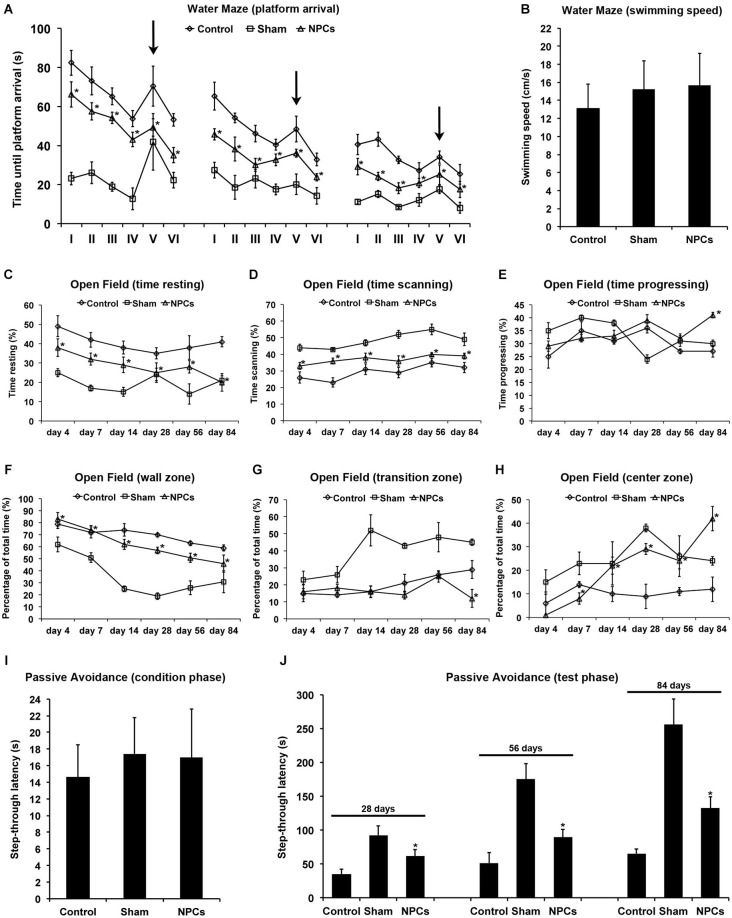
**Neural progenitor cells reduce impairment of learning and memory after mild cerebral ischemia**. Cognitive impairment after 45 min of cerebral ischemia was analyzed using a modified water maze test, the open field test and the passive avoidance test. Mice were given intravenous injection of either neural progenitor cells (“NPCs”; 10^6^ cells in 100 µl of PBS) or PBS (“Control”) 6 h after stroke. Sham animals (“Sham”) underwent the same operation, but without inserting a filament followed by intravenous injection of 100 µl PBS at 6 h. The water maze test **(A)** was performed on day 26–28, day 54–56 and on day 82–84. For each time point, four runs in the morning and four runs in the afternoon with a maximal time of 90 s were performed. Four runs were always grouped as one trial, thus making a total of 6 trials (I–VI) per time point. For runs 1–16 (trials I-IV) and runs 21–24 (trial VI), the platform was always located in the same quadrant. For runs 17–20 (trial V), the platform was re-located within a different quadrant of the water maze pool (“task switch”; indicated by arrows). Maximal testing time was 90 s, and animals that did not reach the platform were scored 90 s. Noteworthy, swimming speed did not differ between the various groups **(B)**. The open field test was used for analysis of both spontaneous post-ischemic locomotor activity and exploration behavior. In order to analyze spontaneous locomotor activity of stroke animals, resting **(C)**, scanning **(D)** and progressing **(E)** times were measured. Exploration behavior and anxiety were assessed via analysis of the percentage of time spent in the wall zone **(F)**, the transition zone **(G)** and the center zone **(H)**. The passive avoidance test was performed using a cage consisting of a large illuminated (“white”) and a smaller non-illuminated (“dark”) compartment, which were separated from each other by a guillotine gate. Animals were detected by means of high sensitivity weight transducers. Natural behavior of the mouse includes escaping the white compartment through the gate, thus entering the small dark compartment. The pre-ischemic condition phase **(I)** depicts the maximal step-through latency when leaving the white compartment towards the dark compartment with a maximal testing time of 50 s. When mice entered the dark compartment, they received an inevitable electric foot shock. During the testing phase **(J)**, mice were again put into the white compartment and the time until animals reached the dark compartment was measured. No shock was applied during the testing phase, and the maximal testing time was 300 s. Animals that did not enter the dark compartment were scored 300 s. All behavioral tests were performed at the time points given as described in the materials and methods section of the manuscript. Number of animals used for statistical analysis was *n* = 15 for sham animals, *n* = 14 for NPC-treated mice and *n* = 16 for controls. * Significantly different from control, *p* < 0.05.

**Figure 5:**
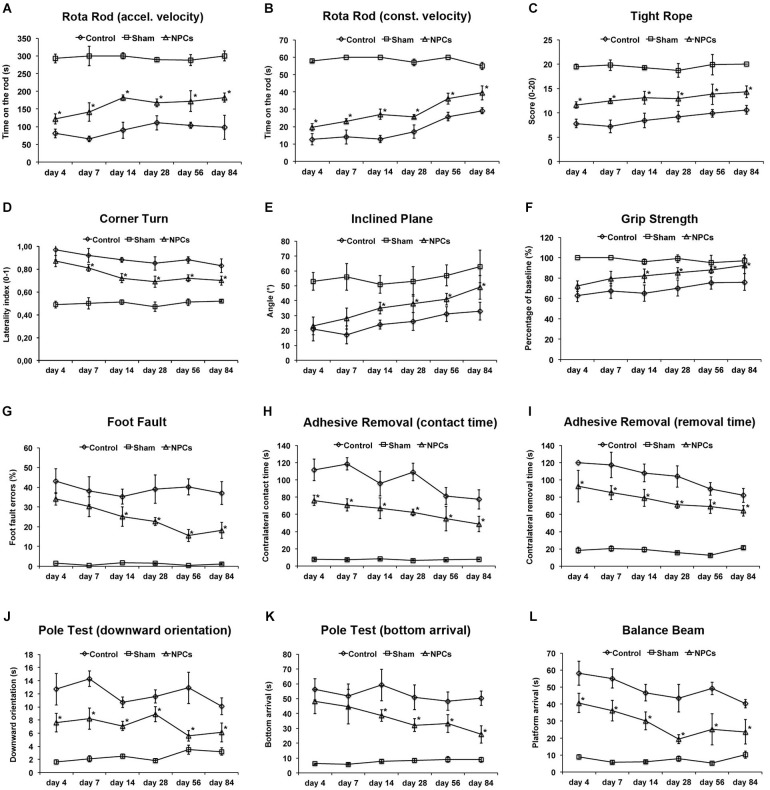
**Neural progenitor cells reduce motor coordination impairment after severe cerebral ischemia**. Animals underwent 90 min of cerebral ischemia followed by a survival period of 84 days. Mice received intravenous injection of either neural progenitor cells (“NPCs”; 10^6^ cells in 100 µl of PBS) or PBS (“Control”) 6 h after stroke. Sham animals (“Sham”) underwent the same operation, but without inserting a filament followed by intravenous injection of 100 µl PBS at 6 h. Behavioral tests included the rota rod test with accelerating **(A)** and constant **(B)** velocity, for which maximal testing time was 300 s **(A)** and 60 s **(B)**. For the tight rope test **(C)**, a validated score was used from 0 (min) to 20 (max). The corner turn test **(D)** was analyzed using the laterality index (number of left turns – number of right turns)/10. For the inclined plain test **(E)**, the angle until the mice was still able to hold its ground was measured and used for statistical analysis, whereas the grip strength **(F)** test analyzed the percentage of force an animal was able to exert on a steel wire. For the foot fault test **(G)**, mice were placed on an elevated steel grid, and the relative number of foot faults of the right (impaired) forelimb was counted. The adhesive removal test included attachment of a defined tape at the paws with subsequent determination of both contact time **(H)** and removal time **(I)**. Maximal testing time of the test was 120 s. The pole test analyzed both downward orientation **(J)** and bottom arrival **(K)** when the mouse was placed head upward on the top of a vertical pole, thus predominantly testing extrapyramidal motor locomotion. Maximal testing time was 60 s for these tests. If the animal was unsuccessful for either task, it was scored 60 s. The balance beam test **(L)** consisted of an apparatus with a 110 cm long beam with constantly reduced width, starting with 12 mm width at the beginning and 5 mm width at the end of the beam. The time (maximal time was 60 s) until the mouse reached the platform at the end of the beam was measured. Maximal testing time was 60 s. The tests were performed at the time points given as described in the materials and methods section of the manuscript. Number of animals used for statistical analysis was *n* = 15 for sham animals, *n* = 16 for NPC-treated mice and *n* = 17 for controls. However, one NPC-treated mouse and four control animals could not perform the tight rope test and the pole test due to the severity of the disease and were excluded from the statistical analysis in these three tests. * Significantly different from control, *p* < 0.05.

### Balance beam test

This test was performed as previously described with slight modification (Luong et al., 2011). For this test, mice were put on a 110 cm long beam with constantly reduced width, i.e., 12 mm width at the beginning and 5 mm width at the end of the beam. The wooden beam was paralleled and 60 cm elevated from the ground on which cushions were placed in order to avoid injury of the animals. A platform was located at the end of the beam and the time measured until the mouse reached that platform. Maximal testing time was 60 s. Animals that failed to reach the platform were scored 60 s. Each animal was tested twice per time point and means were calculated.

### Corner turn test

Two vertical boards were attached to each other forming an angle of 30°. The mouse was put into the apparatus and tested for the side chosen to leave the corner once it made contact to the boards with its whiskers. The amount of trials performed per day was 10. Whereas healthy animals leave the corner without side preference, mice suffering from stroke preferentially leave the corner towards the non-impaired (i.e., left) body side. The laterality index was calculated as follows: (number of left turns – number of right turns)/10.

### Foot fault test

The test was performed according to Rogers et al. with slight modifications (Rogers et al., 1997). Briefly, mice were placed on an elevated steel grid. Thereafter, the total number of steps for each forelimb was counted (i.e., placement of forelimbs on the grid). In the process of moving forward, the foot fault errors (i.e., when the animal’s forelimb was misplaced and thus fell through the grid) were recorded. Data is given as percentage of foot fault errors for the right impaired forelimb referring to the total amount of right forelimb steps.

### Grip strength test

The grip strength was performed as previously described (Kilic et al., 2010; Reitmeir et al., 2011). Briefly, the test includes a spring balance coupled with a Newton meter (Medio-Line Spring Scale, metric, 300 g, Pesola AG, Switzerland) that is attached to a triangular steel wire. Animals were pulled by the tail followed by instinctive grasping of the spring by the animal. Thus, the mouse exerts force on the steel wire, which can be measured as a read out parameter. The test was performed eight times on the right paretic forepaw of mice, whereas the left non-paretic forepaw was wrapped with an adhesive tape. The means were calculated for all eight measurements. For these data, percentage values were calculated with pre-ischemic values set as 100%.

### Inclined plane test

The test was performed as described before (Pajoohesh-Ganji et al., 2010; Popa-Wagner et al., 2010). Briefly, mice were put head down on a platform (40 cm × 60 cm) with a rubber surface followed by a successive increase of the platform angle. The experiment ended when the mouse was no longer able to hold its position for 5 s without sliding down. The last angle that the mouse was able to hold for 5 s was documented. A full experiment consisted of three trials at the time points given with an intertrial break of one minute to allow animals to recover. Means were calculated for each time point and used for statistical analysis.

### Open field test

The test was used to assess both spontaneous post-ischemic locomotor activity and post-ischemic exploration behavior as previously described by our group (Kilic et al., 2008). Briefly, mice where placed into a round arena (diameter of 120 cm) surrounded by 30 cm high sidewalls. The stage consisted of three sections including an outer wall zone, a transition zone and an inner center zone. The mouse was always placed close to the wall and its behavior was analyzed for 10 min using an electronic imaging system (Ethovision XT, Noldusn The Netherlands). In order to analyze spontaneous locomotor activity of stroke animals, resting, scanning and progressing times were measured. Exploration behavior and anxiety were assessed via analysis of the percentage time spent in each of the three zones.

### Passive avoidance test

The test was performed as described before with slight modifications (Bouët et al., 2007). Briefly, mice were first introduced and conditioned to the setting apparatus using a passive avoidance cage (Harvard Apparatus, USA) that consisted of a large illuminated (“white”) and a smaller non-illuminated (“dark”) compartment. These two compartments were separated from each other by a guillotine gate. Animals were detected by means of high sensitivity weight transducers. According to the natural behavior of the rodent, mice initially sought to escape the white compartment through the gate, thus entering the small dark compartment. During the condition phase (twice on days 1–3 before induction of ischemia), the maximal step-through latency, i.e., time allowed for the animals to pass through the gate after having been put farthest away from the gate within the white compartment was 50 s. All mice passed the gate within this time period without significant differences between each other. After having entered the dark compartment, mice received an inevitable electric foot shock (0.4 mA, 2 s). During the testing phase (twice per time point), mice were again put into the white compartment farthest away from the guillotine gate. This time, no shock was applied and the time until the animals entered the dark compartment (“step-through latency”) was monitored. Maximal testing time was 300 s. Animals that did not enter the dark compartment were scored 300 s.

### Pole test

This test was performed with slight modifications according to a previous protocol (Bouët et al., 2007). A vertical pole (60 cm high with rough surface) was used for this test, which predominantly analyzes extrapyramidal motor locomotion. Mice were placed head upward on the top of the pole. Thereafter, both time taken to orientate the body completely downwards and to reach the floor with all four paws were recorded. Maximal testing time was 60 s. If the animal was unsuccessful for either task, it was scored 60 s. Each animal was tested four times at the time points given followed by calculation of means that were used for statistical analysis.

### Rota rod test

The rota rod test was performed using a treadmill with a diameter of 3 cm (TSE Systems, Bad Homburg, Germany). This test was performed with either accelerating velocity (4–40 rpm) or at constant velocity of 40 rpm. For the accelerating test conditions, maximal velocity was achieved after 260 s with a maximal testing time of 300 s. On the contrary, maximal testing time for constant velocity (40 rpm) was 60 s. The time until the animals dropped was measured. Tests were always performed twice and means were used for statistical analysis.

### Tight rope test

This test was performed by means of a 60 cm long rope that was attached to two opposing platforms as previously described by us (Doeppner et al., 2011). The mouse was placed with its forepaws at the middle of the rope. Two parameters were analyzed, i.e., whether or not the animal reached either of the two platforms and the time needed for reaching the platform. The maximal testing time was 60 s. Accordingly, these two parameters formed a validated score between 0 (worst performance) and 20 (best performance). The test was performed twice per time point and means were calculated.

### Water maze test

The water maze test was performed as described before by our group (Doeppner et al., 2013). Briefly, a modified water maze test was performed on days 26–28, days 54–56 and on days 82–84. Each test day consisted of four runs in the morning and four in the afternoon with a maximal time for each run of 90 s. Four runs were always grouped as one trial, thus making a total of 6 trials (I–VI) per time point. For runs 1–16 (trials I–IV) and runs 21–24 (trial VI), the transparent platform was always located in the same quadrant (i.e., upper left quadrant #1). However, for runs 17–20 (trial V) the platform was re-located within a different quadrant of the water maze pool (“task switch”). If an animal did not reach the platform, it was gently guided to the platform and scored 90 s. Both swimming speed and time needed to reach the platform were tracked using a computer-based video camera system (TSE Systems, Germany). Data are given as means per trial. Finally, relative mean times spent in quadrant #1 out of trials I–IV and trial VI was determined per time point, thus indicating spatial learning of experimental mice.

### Statistical analysis

The data are given as means ± standard deviation (SD). A one-way analysis of variance (ANOVA) followed by the Tukey’s *post hoc* test was performed for statistical analysis. A *p* value of < 0.05 was considered to be statistically significant.

## Results

### NPCs induce acute neuroprotection after both mild and severe cerebral ischemia

Before using various behavioral tests in order to analyze post-ischemic functional recovery after treatment with NPCs, we first performed a proof of concept experiment indicating whether or not NPCs induce neuroprotection in our experimental paradigms. Using an intravenous cell delivery route with a transplantation of 10^6^ NPCs at 6 h post-stroke, a time point that has been previously shown to be beneficial after a 45-min stroke (Doeppner et al., 2012a), we analyzed infarct volumes and brain injury. Noteworthy, the amount of intracerebral GFP^+^ NPCs was in the same order with typical orientation within the ischemic lesion site as previously described (Doeppner et al., 2012a). Two days after stroke, we observed 89.7 ± 17.3 NPCs per mm^2^ after 45 min of cerebral ischemia and 76.2 ± 25.1 NPCs per mm^2^ after 90 min of cerebral ischemia. Analysis of infarct volumes revealed a significant reduction on day 2 after induction of both mild (45 min) and severe (90 min) cerebral ischemia (Figures 2A,B). In line with the latter, the number of TUNEL^+^ cells (Figure 2C) and the amount of microglial cells (Figure 2D) were significantly reduced after NPC treatment when compared to PBS controls. Thus, systemic transplantation of NPCs induces significant acute neuroprotection after both mild and severe cerebral ischemia, providing the basis for enhanced functional recovery due to cell based therapy.

### NPCs reduce motor coordination deficits after mild cerebral ischemia

Although enhanced post-stroke functional recovery due to NPC treatment has been described before, the most appropriate behavioral tests for these circumstances yet have to be defined. As a matter of fact, a great deal of behavioral tests exists for mice under various physiological and pathological circumstances, but systematic analysis of these tests in one condition is scarce. In order to systematically analyze appropriate motor coordination tests for assessment of post-stroke functional impairment after NPC treatment, we used nine well-known behavioral tests. As shown by our group before, NPC treatment after 45 min of stroke results in better test performance in the rota rod test, the tight rope test and the corner turn test (Figures 3A–D). Since accelerating velocity in the rota rod test may lead to exhaustion of stroke mice, we also applied a constant velocity paradigm with a testing time of only 60 s. However, there was no difference in the test results, i.e., NPC-treated mice scored better under both experimental paradigms. Noteworthy, test performance of treated mice approximated sham (non-ischemic) animals over time in the aforementioned tests. On the contrary, we did not observe any significant differences after NPC treatment using the inclined plane test and the grip strength test (Figures 3E,F). Further analysis of motor coordination deficits after 45 min of cerebral ischemia revealed significantly improved outcome after NPC treatment in the foot fault test, the adhesive removal test, the pole test and the balance beam test (Figures 3G–L). Noteworthy, contact time and removal time of the ipsilateral forepaw as assessed by the adhesive removal test were also significantly affected by NPC treatment (Figure S1). These data indicate enhanced functional recovery of stroke after NPC treatment. Nevertheless, the inclined plane and the grip strength test are not recommended for assessment of motor coordination deficits under mild cerebral ischemia.

### NPCs reduce cognitive impairment after mild cerebral ischemia

Occlusion of the middle cerebral artery in the mouse model usually does not include injury of the hippocampal formation. Nevertheless, reduced test scores and/or cognitive impairment such as for the water maze test also do occur after injury of both basal ganglia and cortical areas (Bingham et al., 2012). We therefore analyzed post-ischemic impairment of learning and memory using the water maze test, the open field test and the passive avoidance test. Using the water maze test, NPC-treated animals significantly scored better than controls, albeit improved test performance over time was observed under all conditions (Figure 4A). Noteworthy, swimming speed did not significantly differ between experimental groups (Figure 4B). In line with this, the relative amount of time spent in quadrant #1 where the platform was located for trials I–IV and trial VI was significantly increased in mice that had been treated with NPCs. En detail, NPC-treated mice spent 48.2 ± 6.7% (day 26–28), 62.7 ± 9.8% (day 54–56) and 75.6 ± 11.4% (day 82–84) of total time in quadrant #1. On the contrary, controls spent 34.1 ± 5.6% (day 26–28), 42.7 ± 3.9% (day 54–56) and 56.2 ± 6.3% (day 82–84) of total time within the platform containing quadrant #1. These data thus suggest that the water maze test is suitable for assessing learning and memory capabilities after mild cerebral ischemia.

Not necessarily limited to post-ischemic cognitive impairment, further analysis regarding spontaneous locomotor activity and exploration behavior was done using the open field test. Locomotor activity was assessed via analysis of time stroke animals spent with resting (Figure 4C), scanning (Figure 4D) or progressing (Figure 4E) within the open field. As such, treatment with NPCs significantly reduced the relative amount of time mice spent with resting, suggesting an increased spontaneous locomotor activity in these animals. In line with this, NPC treatment resulted in stroke mice spending more time in the non-protected center zone of the open field when compared to controls (Figures 4F–H). Consequently, treatment with NPCs increased exploration behavior and reduced anxiety in these animals.

The passive avoidance test is a fear associated means to study learning and memory, using an aversive stimulus like a foot shock when mice follow their natural behavior such as entering the dark compartment. After the pre-ischemic condition phase that showed no significant difference between the experimental groups (Figure 4I), mice were exposed to the test setting at the time points given. Step through latencies, i.e., the latency until mice passed through the gate into the dark chamber according to their initial natural behavior, were highest in sham-operated mice. However, treatment with NPCs also resulted in significantly increased step through latencies when compared to non-treated controls (Figure 4J). These results suggest enhanced learning and memory patterns due to NPC-treatment after mild cerebral ischemia; all of which can be analyzed by the aforementioned three tests under these conditions.

### Systemic transplantation of NPCs reduces motor coordination deficits after severe cerebral ischemia

We next addressed the question whether or not systemic transplantation of NPCs also yielded long-term functional recovery as assessed by the aforementioned behavioral tests on motor coordination. As shown for mild cerebral ischemia (Figures 3A–D), transplantation of NPCs resulted in improved functional recovery of stroke animals when compared to controls in the rota rod, the tight rope and the corner turn test (Figures 5A–D). However, restrictions apply for the tight rope test that is a strenuous task, thus making it impossible for one NPC-treated mouse and four controls to actually perform the test. Instead of scoring these animals with a minimum score (i.e., 0), these mice were excluded from statistical analysis of the tight rope test. As for the inclined plane, the grip strength test, the foot fault test, the adhesive removal test, the pole test and the balance beam test (Figures 5E–L), NPC-treated mice always scored better than controls. Regarding the adhesive removal test, contact time and removal time of the ipsilateral forepaw were again significantly affected by NPC treatment (Figure S1). Noteworthy, the same restrictions applied for the pole test as for the aforementioned tight rope test, suggesting that both the tight rope test and the pole test are not suitable for behavioral analysis after severe cerebral ischemia.

### Cognitive impairment after severe cerebral ischemia is reduced by NPC transplantation

**Figure 6:**
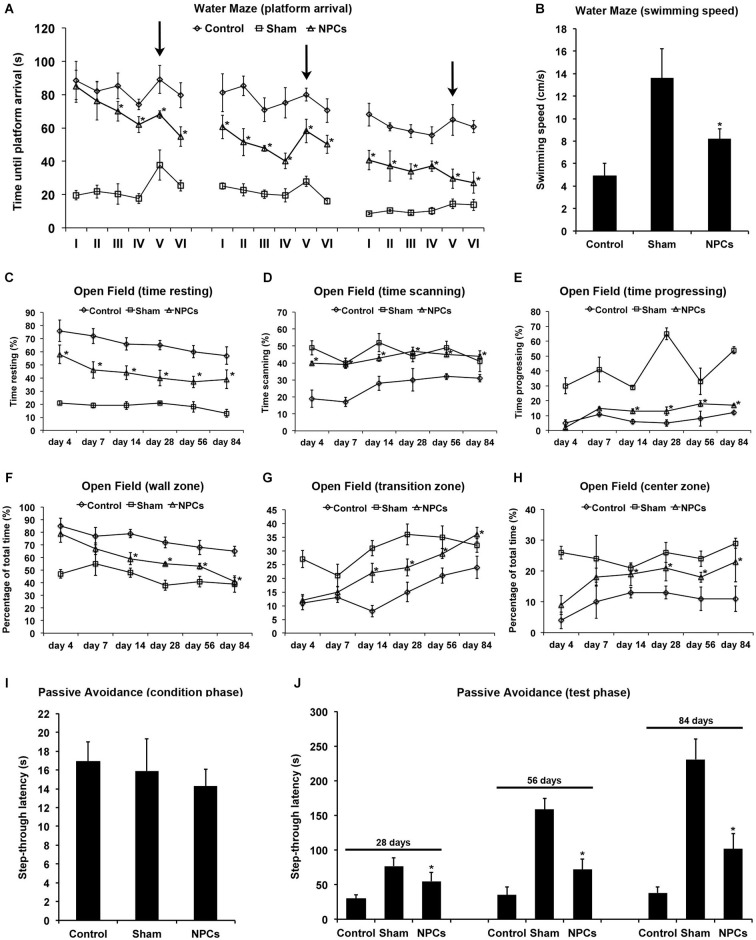
**Neural progenitor cells reduce impairment of learning and memory after severe cerebral ischemia**. Cognitive impairment after 90 min of cerebral ischemia was analyzed using the water maze test, the open field test and the passive avoidance test. Intravenous injections with either neural progenitor cells (“NPCs”; 10^6^ cells in 100 µl of PBS) or PBS (“Control”) were performed at 6 h after stroke. Sham animals (“Sham”) underwent the same operation, but without inserting a filament followed by intravenous injection of 100 µl PBS at 6 h after intervention. The water maze test **(A)** was performed on day 26–28, day 54–56 and on day 82–84 with four runs in the morning and four runs in the afternoon per test day. Maximal testing time was 90 s, and animals that did not reach the platform were scored 90 s. Four runs were always grouped as one trial, thus making a total of 6 trials (I–VI) per time point. For runs 1–16 (trials I–IV) and runs 21–24 (trial VI), the platform was always located in the same quadrant. For runs 17–20 (trial V), the platform was re-located within a different quadrant of the water maze pool (“task switch”; arrows). Swimming speed **(B)** is given as means out of all runs per condition. The open field test was used for analysis of both spontaneous post-ischemic locomotor activity and exploration behavior. In order to analyze spontaneous locomotor activity of stroke animals, resting **(C)**, scanning **(D)** and progressing **(E)** times were analyzed. Exploration behavior and anxiety were assessed via analysis of the percentage of time spent in the wall zone **(F)**, the transition zone **(G)** and the center zone **(H)**. The passive avoidance test was performed using a cage consisting of a large illuminated (“white”) and a smaller non-illuminated (“dark”) compartment, which were separated from each other by a guillotine gate. Natural behavior of the mouse includes escaping the white compartment through the gate, thus entering the small dark compartment. The pre-ischemic condition phase **(I)** depicts the maximal step-through latency when leaving the white compartment towards the dark compartment with a maximal testing time of 50 s. When mice entered the dark compartment, they received an inevitable electric foot shock. During the testing phase **(J)**, mice were again put into the white compartment and the time until animals reached the dark compartment was measured. No shock was applied during the testing phase, and the maximal testing time was 300 s. Animals that did not enter the dark compartment were scored 300 s. All behavioral tests were performed at the time points given as described in the materials and methods section of the manuscript. Number of animals used for statistical analysis was *n* = 15 for sham animals, *n* = 16 for NPC-treated mice and *n* = 17 for controls. However, one NPC-treated mouse and five control animals could not perform the water maze test due to the severity of the disease and would have drowned during the behavioral test, if the test had not been canceled. These mice were excluded from the statistical analysis of the water maze test, thus ending up with 15 sham animals, 15 NPC-treated mice and 12 control mice. * Significantly different from control, *p* < 0.05.

Water maze test performance including the task switch was significantly increased for as long as 84 days when mice received systemic injections of NPCs 6 h after induction of severe cerebral ischemia (Figure 6A). However, swimming speed of NPC-treated mice significantly differed from controls (Figure 6B), whereas the relative amount of time spent in the correct quadrant #1 did not differ from each other (data not shown). Thus, water maze test performance of NPC-treated mice after severe cerebral ischemia was significantly affected by reduced motor coordination deficits (Figure 5) of these animals. In line with this, some animals (one NPC-treated mouse and five controls) were not able to perform the water maze test due to the severity of brain injury and would have drowned, if the test had been completed. These mice were not included for final statistical analysis. Motor coordination thus significantly affected water maze test performance independent of cognitive impairment of stroke animals.

Analysis of the open field test revealed significantly increased spontaneous locomotor activity when mice were given NPCs as compared to controls (Figures 6C–E). Transplantation of NPCs resulted in a reduced relative amount of time that mice spent with resting, whereas scanning and progressing in these mice was increased. Likewise, NPC treatment yielded reduced anxiety as these mice significantly spent more time in the transition and center zone of the open field than controls (Figures 6F–H). Fear associated learning and memory impairment as assessed in the passive avoidance test (Figures 6I–J) was also significantly reduced in NPC-treated mice as indicated by increased step through latencies. These results suggest enhanced learning and memory capabilities due to NPC-treatment after severe cerebral ischemia, albeit the water maze test is not appropriate for this experimental paradigm.

### NPCs reduce post-ischemic brain injury after mild and severe cerebral ischemia

**Figure 7:**
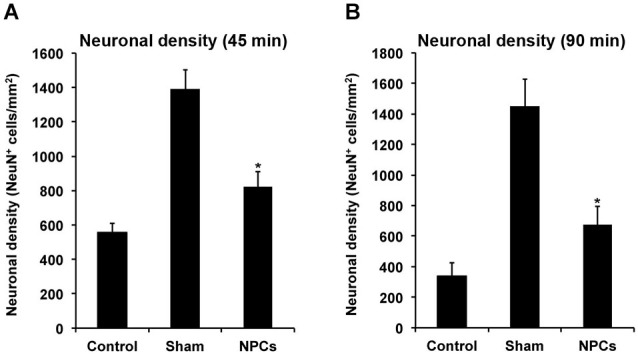
**Neural progenitor cells induce long-term neuroprotection after both mild and severe cerebral ischemia**. Mice underwent 45 min **(A)** or 90 min **(B)** of cerebral ischemia followed by intravenous injection of neural progenitor cells (“NPCs”) or PBS (“Control”) 6 h post-stroke. Sham animals (“Sham”) underwent the same operational technique, but without inserting a filament for occlusion of the middle cerebral artery. Shams received intravenous injection of PBS at 6 h after stroke. Neuronal density was assessed on day 84 via NeuN staining. The number of animals used for statistical analysis after mild cerebral ischemia was *n* = 15 (sham), *n* = 14 (NPC-treated mice) and *n* = 16 (controls), whereas for the severe cerebral ischemia paradigm *n* = 15 (sham), *n* = 16 (NPC-treated mice) and *n* = 17 (controls) was used. * Significantly different from control, *p* < 0.05.

Since improved functional outcome does not necessarily reflect reduction of brain injury and vice versa, neuronal densities were assessed in the long run using NeuN staining (Figure 7). As such, neuronal densities were significantly increased on day 84 after both mild and severe cerebral ischemia when mice were treated with NPCs. These data suggest that the aforementioned improved motor coordination as well as learning and memory capabilities due to treatment of NPCs are associated with reduced histological brain injury in these animals.

## Discussion

The present work systematically analyzed post-ischemic functional impairment after intravenous NPC transplantation with respect to motor coordination and cognitive impairment in mice. Although recent findings by our group suggest that NPC-induced functional recovery is independent of cell delivery timing (Doeppner et al., 2014), we have chosen an experimental paradigm used in previous studies (Doeppner et al., 2012a) that involves acute NPC grafting with subsequent reduction of brain injury. Emphasis during the three-month observation period was not only put on reliability and validity but also on feasibility and cost-effective considerations of the behavioral test in question.

Since motor coordination scores significantly depend on severity of focal cerebral ischemia (Rogers et al., 1997), we induced both mild and severe cerebral ischemia in mice. A great deal of motor coordination tests exists in rodents for physiological and pathological conditions (Bouët et al., 2007; Brooks and Dunnett, 2009; Balkaya et al., 2012), with some of which successfully used by our group in the past (Doeppner et al., 2009a,b, 2010, 2011, 2012a,b).

The rota rod test, which has been first described in 1957 using a drum with constant velocity (Dunham and Miya, 1957), is an excellent test for assessing motor coordination. As mentioned afore, NPCs reduced motor coordination impairment after both mild and severe cerebral ischemia. Although accelerating velocity might involve the risk of exhausting mice that suffered from severe ischemia, test results were reliable and valid using both constant and accelerating velocity paradigms (Dunham and Miya, 1957; Jones and Roberts, 1968). Despite the fact that the rota rod offers the advantage of producing automated data, one has to keep in mind that running on the drum might also involve anxiolytic behavior of the test mice independent of motor coordination deficits (Salam et al., 2009). In line with the aforementioned results of the rota rod test, assessment of sensorimotor deficits in the corner turn test showed significantly better outcome for NPC-treated mice under each experimental paradigm. Initially developed by Zhang et al. (2002), this test provides an excellent stage for analyzing post-stroke motor coordination deficits without depending on the strength of the test mice. Enhanced motor coordination recovery after treatment with NPCs was also observed after both mild and severe cerebral ischemia using the tight rope test. Originally described before as the wire hang test (Gerlai et al., 2000), this test was modified using a test score that pays attention to two parameters, i.e., reaching the platform and time spent on the rope (Doeppner et al., 2011). As such, the test proves very useful for elaborate analyses of post-stroke motor coordination deficits. However, limitations occur when animals are severely affected by stroke or other diseases, thus making them too weak to actually perform this strenuous test. This was the case after severe cerebral ischemia in the present study, resulting in some animals not able to perform the test at all.

The grip strength test and the inclined plane test are well-known tests for assessing motor coordination in rodents both after stroke and other diseases alike (Buchhold et al., 2007; Kilic et al., 2010; Popa-Wagner et al., 2010; Reitmeir et al., 2011). However, both grip strength test and inclined plane test only showed significant differences under conditions of severe cerebral ischemia; no effect of NPC treatment was observed after mild cerebral ischemia. The reason for the latter is that these tests do not only depend on motor coordination activity but also on sheer force of the test mouse, which is not necessarily reduced after mild cerebral ischemia. In this sense, both tests are less sensitive in settings of mild cerebral ischemia.

Further analysis of motor coordination deficits after both mild and cerebral ischemia revealed NPC-induced increased functional recovery using the foot fault, the adhesive removal, the pole test and the balance beam test. The foot fault test (aka as grid walking test) is a well-established and easy to use test for assessment of motor coordination deficits after stroke in rodents (Rogers et al., 1997; Chen et al., 2005; Salam et al., 2009). Noteworthy, the present data shows significant differences between control and NPC-treated mice during the complete observation period of three months, whereas former studies failed to show stroke-induced functional impairment in the foot fault test beyond a time point of 60 days (Zhang et al., 2002). Likewise, both the adhesive removal test and the balance beam test have been successfully used in stroke models (Combs and D’Alecy, 1987; Modo et al., 2000; Li et al., 2001). As for the adhesive removal test, this test even proved to be superior and more feasible than other behavioral tests used for analysis of long-term functional outcome in rodents after stroke (Freret et al., 2009). However, constant testing environments such as testing within the animal’s cage are necessary in order to retrieve reliable and valid data in the adhesive removal test (Schallert and Woodlee, 2005), as should be the case for any other behavioral test as well. Similar findings were found regarding the balance beam test, which was more sensitive in assessment of motor coordination deficits than the rota rod test, albeit these tests were performed in a non-stroke model (Stanley et al., 2005). On the contrary, the pole test cannot be recommended for conditions of severe stroke since some animals are not able to perform the test at all due to the strenuous nature of the task, as has been discussed afore regarding the tight rope test.

Assessment of post-stroke cognitive impairment and therapeutic effects of NPC grafting was performed using the water maze test, the passive avoidance test and the open field test. The water maze test has long been characterized as a reliable means to analyze learning and memory under various conditions including cerebral ischemia in rodents (Morris, 1984; Olsen et al., 1994; Dahlqvist et al., 2004; Winter et al., 2004; Tsai et al., 2011). As stated afore, post-ischemic water maze test performance is also affected after cerebral infarction within the MCA territory (Bingham et al., 2012). Cognitive impairment after mild cerebral ischemia and NPC-induced effects were reliably measured in the water maze test. However, the water maze test is not suitable for detection of cognitive dysfunction after severe cerebral ischemia, since the latter too much includes significantly affected motor coordination as indicated by reduced swimming speed in the test. On the contrary, the passive avoidance test can also be performed on mice suffering from severe stroke and thus under conditions with great motor coordination impairment. In this sense, improved test performance after both mild and severe cerebral ischemia was observed after NPC transplantation. Yet, technical equipment for this test is expensive as is the video tracking system used for the water maze test, thus reducing the feasibility of both tests. The open field test is a simple test for behavioral analysis of rodents, although the test is not exclusively limited to analysis of cognitive function. In the present study, improved test performance after NPC transplantation was observed after both mild and severe cerebral ischemia. In light of its simplicity and cost-efficacy, this test is a reliable and feasible test for studying the aforementioned parameters in stroke mice.

In conclusion, the present study systematically analyzed functional impairment after both mild and severe cerebral ischemia and the effect of acute systemic transplantation of NPCs for as long as three months using a battery of twelve behavioral tests. In terms of reliability and cost-effectiveness considerations, results from our study recommend the use of the corner turn, foot fault, balance beam, and open field test, which do not depend on durations of cerebral ischemia. The study thus provides suggestions for the use of further behavioral tests, which aim to assess functional recovery after stem cell or progenitor cell grafting in stroke models.

## Conflict of interest statement

The authors declare that the research was conducted in the absence of any commercial or financial relationships that could be construed as a potential conflict of interest.
