# Supplementary material for: Effects of neural progenitor cells on post-stroke neurological impairment—a detailed and comprehensive analysis of behavioral tests
Source: Front Cell Neurosci. 2014 Oct 22;8:338. doi: 10.3389/fncel.2014.00338 (PMC4205824; doi:10.3389/fncel.2014.00338)
Supplement: Supplementary file 1 [file Image_1.PDF]

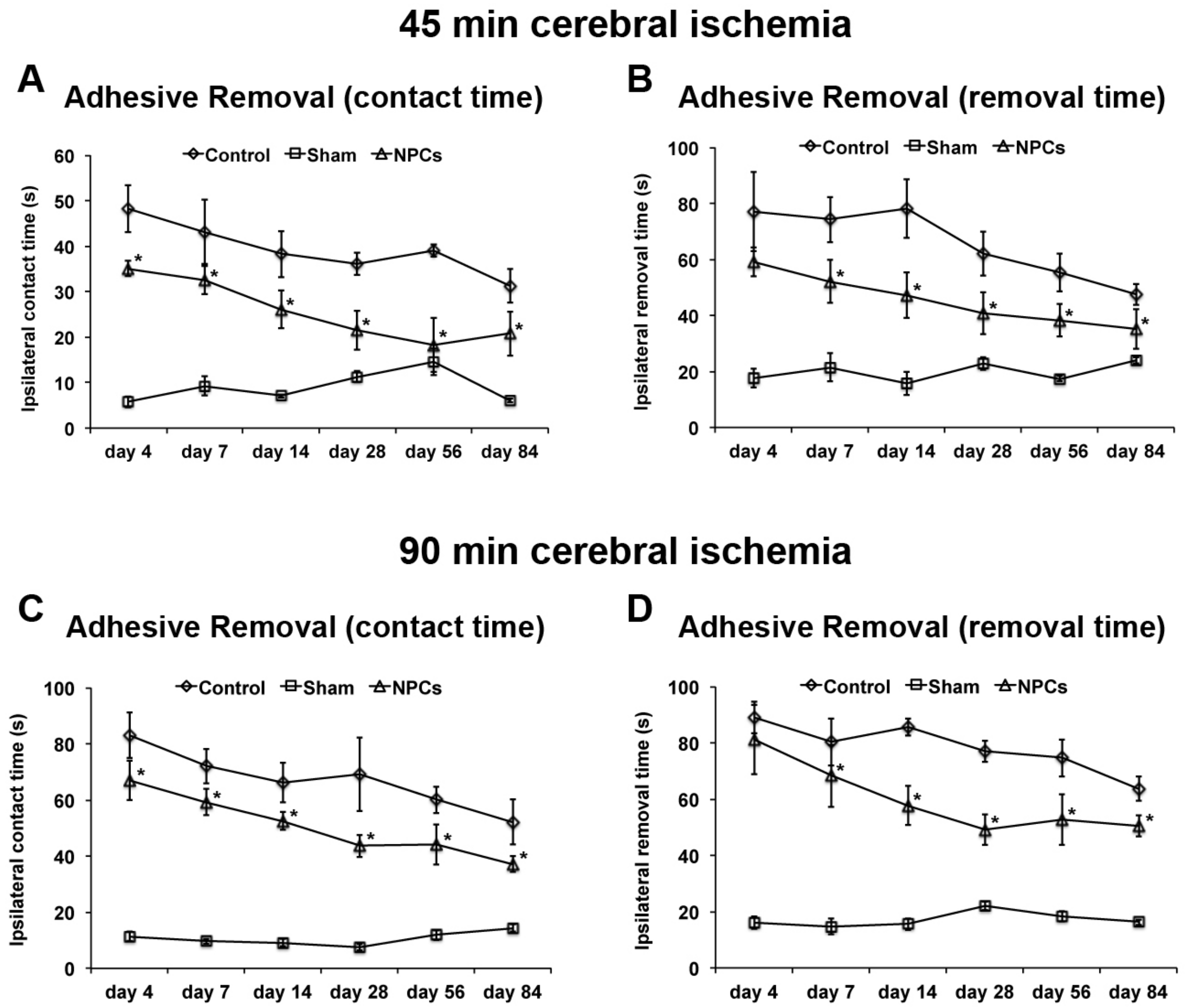

Supplementary Fig. S1. Neural progenitor cells affect adhesive test performance of the ipsilateral forepaw. Since tape removal does not solely depend on the coordination of the mouth and the forepaw on which the tape is attached but also on the skills of the other forepaw, both contact time and removal time for the ipsilateral non-affected forepaw were analyzed. Mice were exposed to either 45 min (A-B) or 90 min of cerebral ischemia (C-D). Treatment paradigm was as follows. Six hours after stroke, mice received intravenous injections of either neural progenitor cells (“NPCs”) or PBS (“Control”), whereas sham animals (“Sham”) underwent the same surgery procedure but without actual thread insertion followed by intravenous infusion of PBS. Survival period was 84 days. Maximal testing time was 120 s. Note that ipsilateral test scores were always better than test scores for the contralateral (i.e. affected) forepaw (Fig. 3 and Fig. 5).
